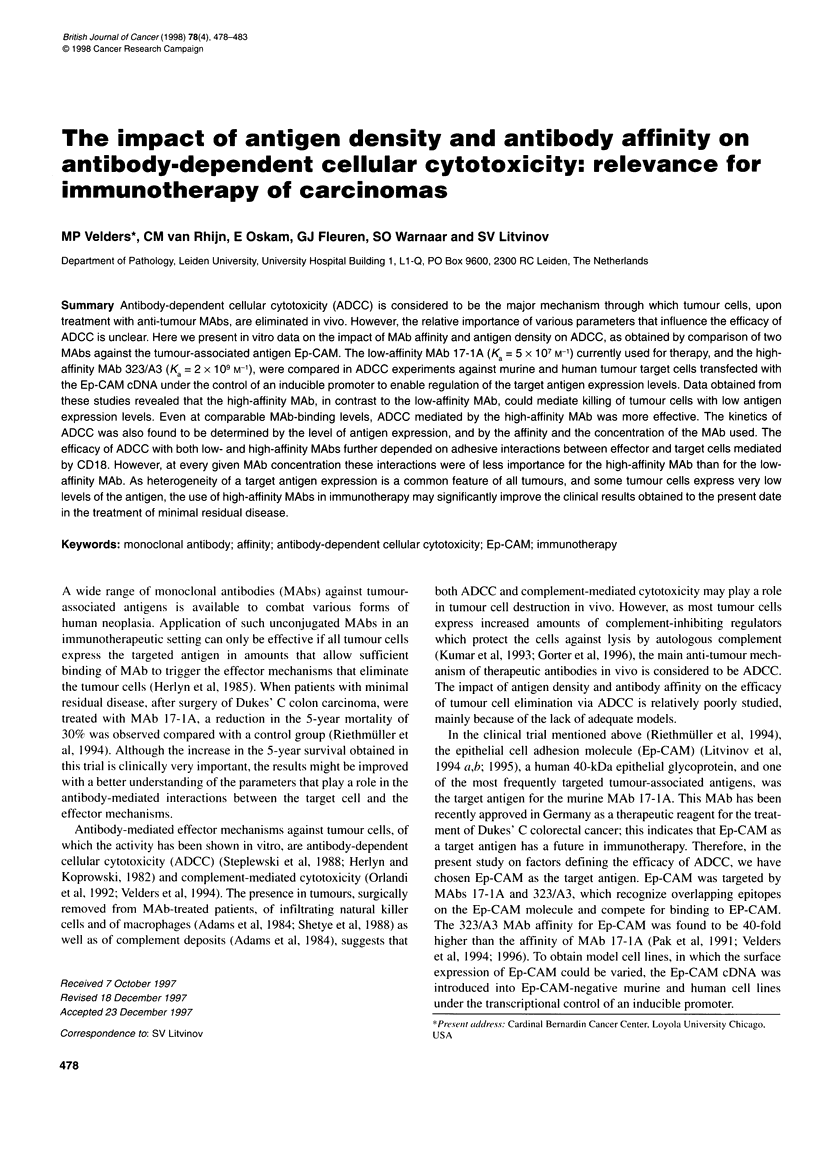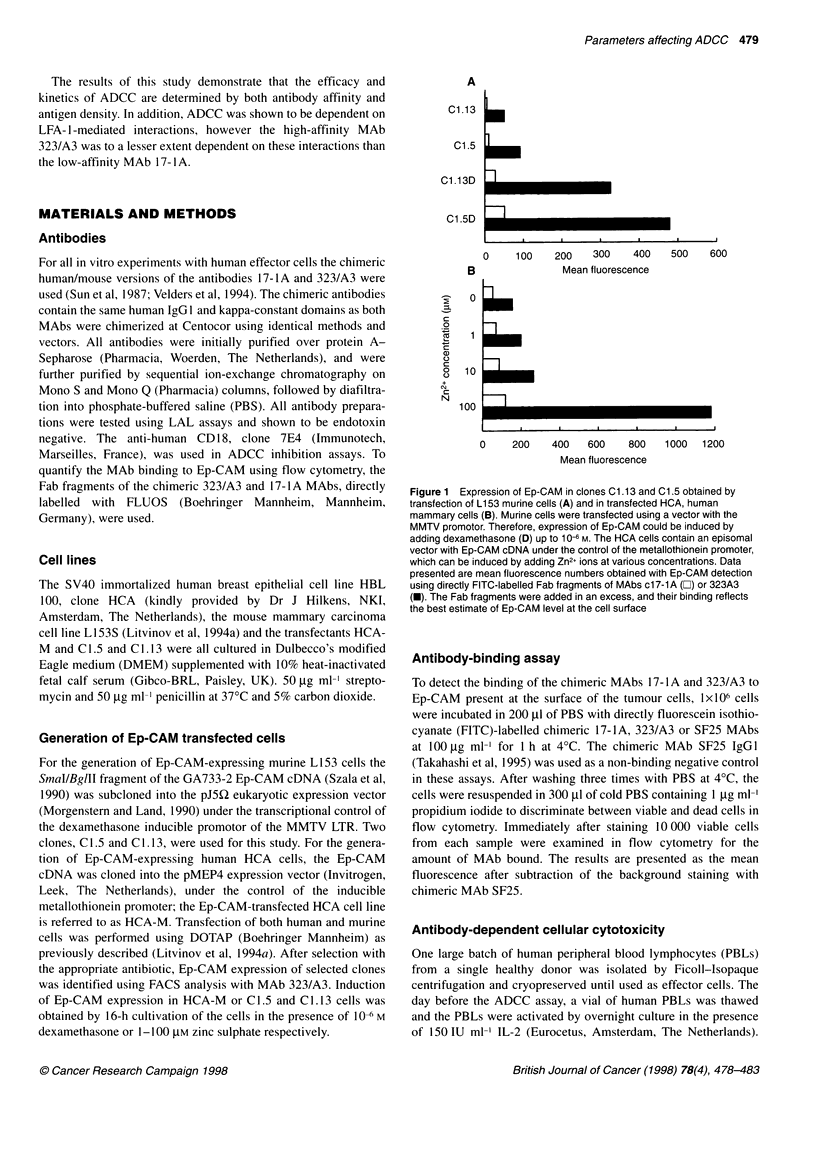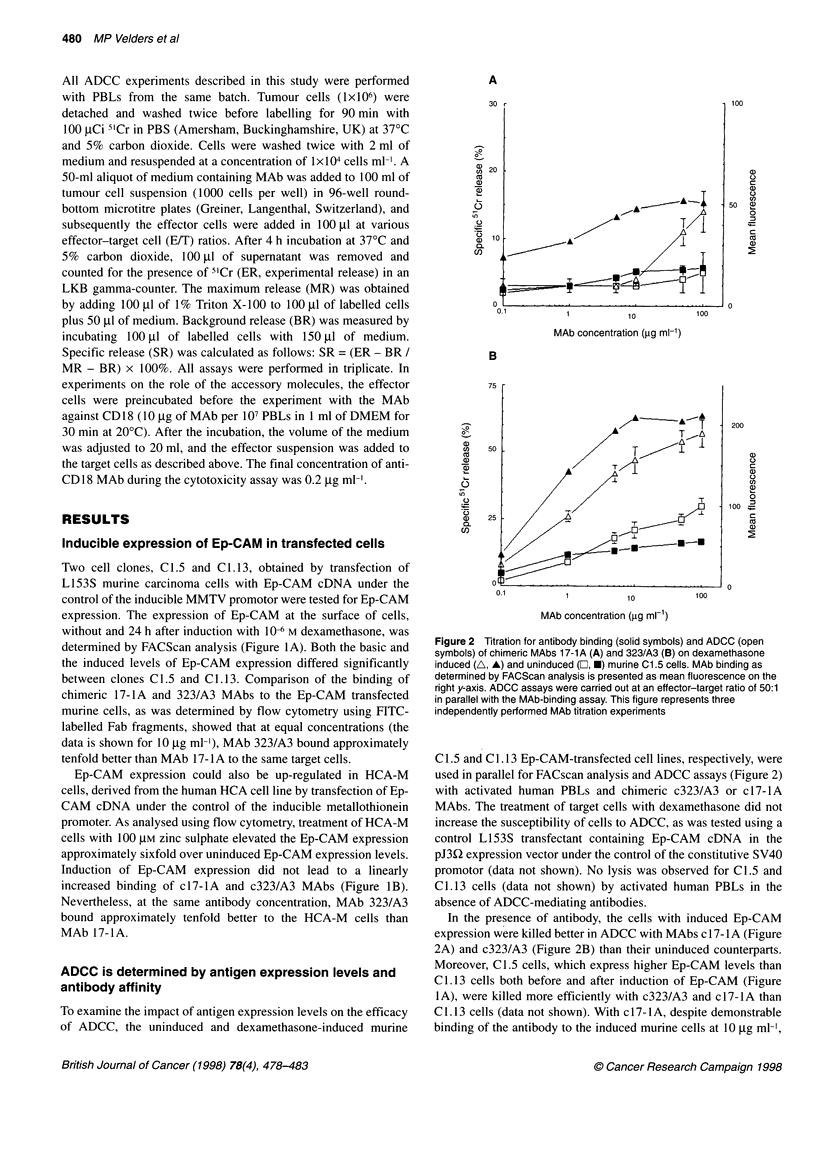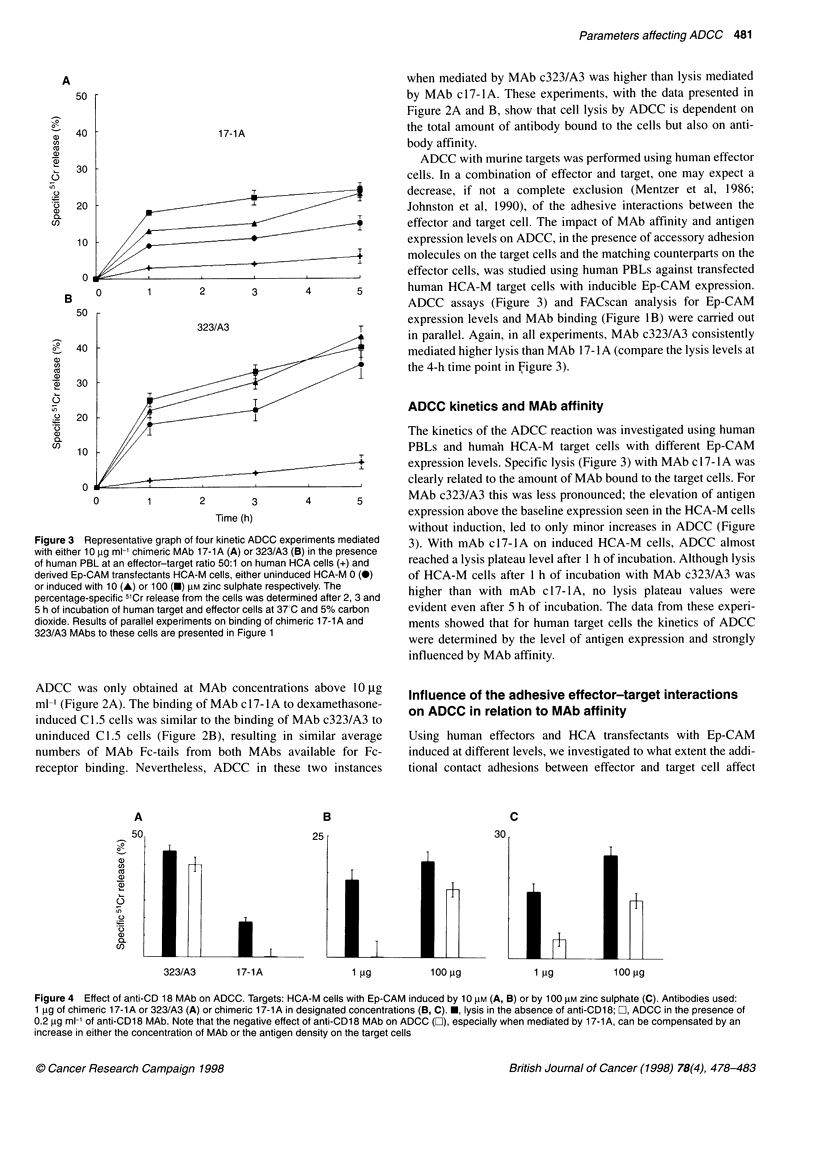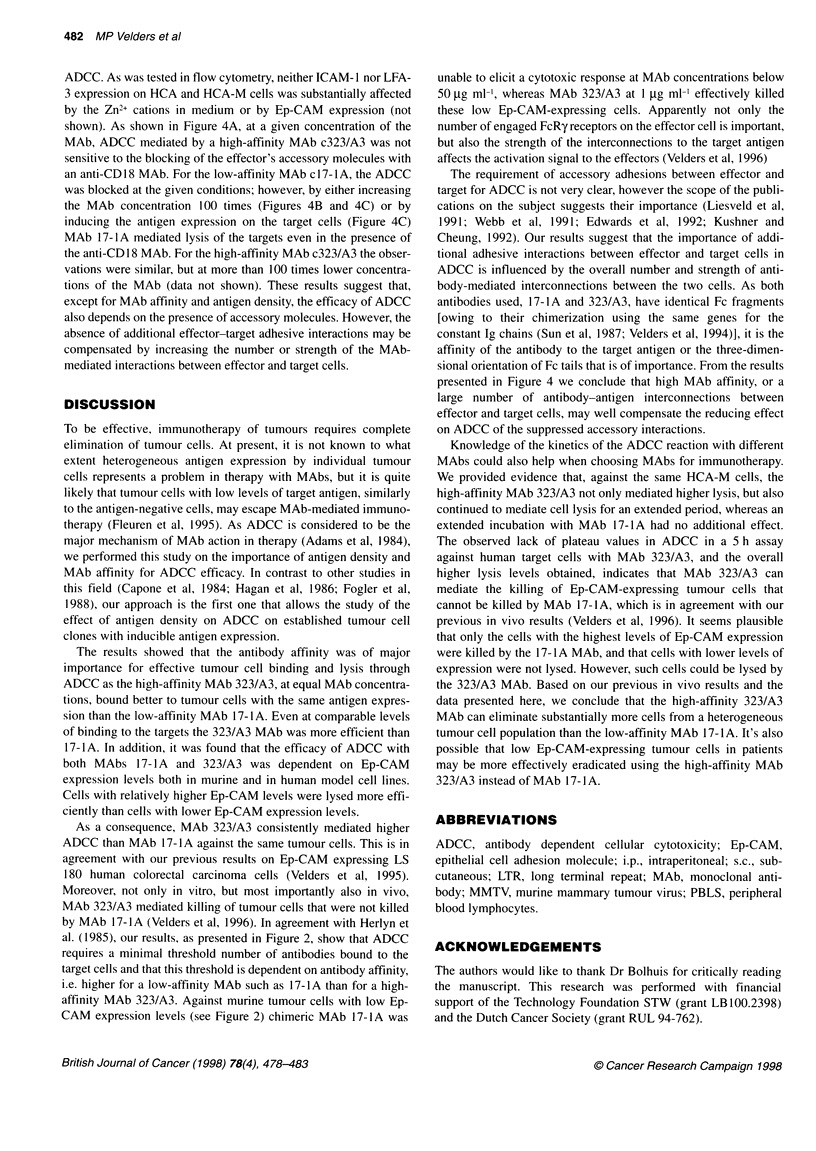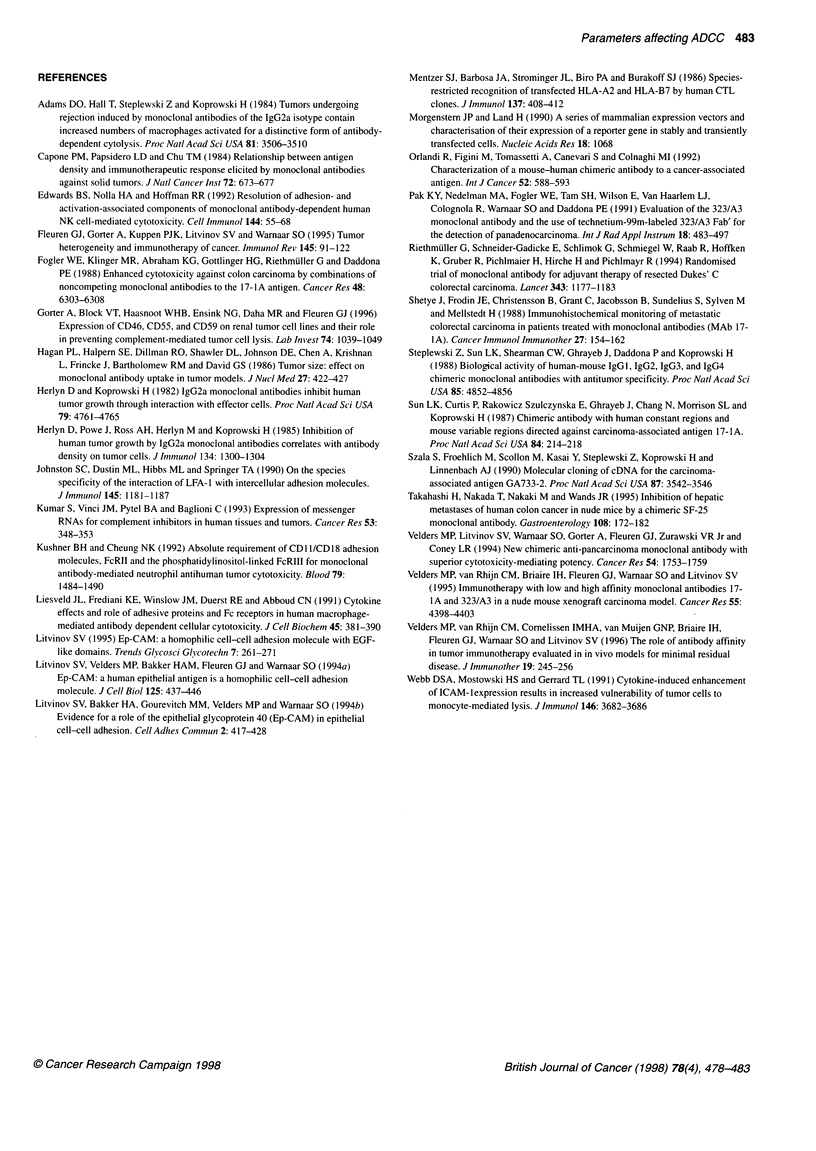# The impact of antigen density and antibody affinity on antibody-dependent cellular cytotoxicity: relevance for immunotherapy of carcinomas.

**DOI:** 10.1038/bjc.1998.518

**Published:** 1998-08

**Authors:** M. P. Velders, C. M. van Rhijn, E. Oskam, G. J. Fleuren, S. O. Warnaar, S. V. Litvinov

**Affiliations:** Department of Pathology, Leiden University, The Netherlands.

## Abstract

Antibody-dependent cellular cytotoxicity (ADCC) is considered to be the major mechanism through which tumour cells, upon treatment with anti-tumour MAbs, are eliminated in vivo. However, the relative importance of various parameters that influence the efficacy of ADCC is unclear. Here we present in vitro data on the impact of MAb affinity and antigen density on ADCC, as obtained by comparison of two MAbs against the tumour-associated antigen Ep-CAM. The low-affinity MAb 17-1A (Ka = 5 x 10(7)M(-1)) currently used for therapy, and the high-affinity MAb 323/A3 (Ka = 2 x 10(9) M(-1)), were compared in ADCC experiments against murine and human tumour target cells transfected with the Ep-CAM cDNA under the control of an inducible promoter to enable regulation of the target antigen expression levels. Data obtained from these studies revealed that the high-affinity MAb, in contrast to the low-affinity MAb, could mediate killing of tumour cells with low antigen expression levels. Even at comparable MAb-binding levels, ADCC mediated by the high-affinity MAb was more effective. The kinetics of ADCC was also found to be determined by the level of antigen expression, and by the affinity and the concentration of the MAb used. The efficacy of ADCC with both low- and high-affinity MAbs further depended on adhesive interactions between effector and target cells mediated by CD18. However, at every given MAb concentration these interactions were of less importance for the high-affinity MAb than for the low-affinity MAb. As heterogeneity of a target antigen expression is a common feature of all tumours, and some tumour cells express very low levels of the antigen, the use of high-affinity MAbs in immunotherapy may significantly improve the clinical results obtained to the present date in the treatment of minimal residual disease.


British Journal of Cancer (1998) 78(4), 478–483
© 1998 Cancer Research Campaign

### The impact of antigen density and antibody affinity on antibody-dependent cellular cytotoxicity: relevance for immunotherapy of carcinomas

**MP Velders*, CM van Rhijn, E Oskam, GJ Fleuren, SO Warnaar and SV Litvinov**

Department of Pathology, Leiden University, University Hospital Building 1, L1-Q, PO Box 9600, 2300 RC Leiden, The Netherlands

**Summary** Antibody-dependent cellular cytotoxicity (ADCC) is considered to be the major mechanism through which tumour cells, upon treatment with anti-tumour MAbs, are eliminated in vivo. However, the relative importance of various parameters that influence the efficacy of ADCC is unclear. Here we present in vitro data on the impact of MAb affinity and antigen density on ADCC, as obtained by comparison of two MAbs against the tumour-associated antigen Ep-CAM. The low-affinity MAb 17-1A ($K_a = 5 \times 10^7$ M$^{-1}$) currently used for therapy, and the high-affinity MAb 323/A3 ($K_a = 2 \times 10^9$ M$^{-1}$), were compared in ADCC experiments against murine and human tumour target cells transfected with the Ep-CAM cDNA under the control of an inducible promoter to enable regulation of the target antigen expression levels. Data obtained from these studies revealed that the high-affinity MAb, in contrast to the low-affinity MAb, could mediate killing of tumour cells with low antigen expression levels. Even at comparable MAb-binding levels, ADCC mediated by the high-affinity MAb was more effective. The kinetics of ADCC was also found to be determined by the level of antigen expression, and by the affinity and the concentration of the MAb used. The efficacy of ADCC with both low- and high-affinity MAbs further depended on adhesive interactions between effector and target cells mediated by CD18. However, at every given MAb concentration these interactions were of less importance for the high-affinity MAb than for the low-affinity MAb. As heterogeneity of a target antigen expression is a common feature of all tumours, and some tumour cells express very low levels of the antigen, the use of high-affinity MAbs in immunotherapy may significantly improve the clinical results obtained to the present date in the treatment of minimal residual disease.

**Keywords:** monoclonal antibody; affinity; antibody-dependent cellular cytotoxicity; Ep-CAM; immunotherapy

A wide range of monoclonal antibodies (MAbs) against tumour-associated antigens is available to combat various forms of human neoplasia. Application of such unconjugated MAbs in an immunotherapeutic setting can only be effective if all tumour cells express the targeted antigen in amounts that allow sufficient binding of MAb to trigger the effector mechanisms that eliminate the tumour cells (Herlyn et al, 1985). When patients with minimal residual disease, after surgery of Dukes' C colon carcinoma, were treated with MAb 17-1A, a reduction in the 5-year mortality of 30% was observed compared with a control group (Riethmüller et al, 1994). Although the increase in the 5-year survival obtained in this trial is clinically very important, the results might be improved with a better understanding of the parameters that play a role in the antibody-mediated interactions between the target cell and the effector mechanisms.

Antibody-mediated effector mechanisms against tumour cells, of which the activity has been shown in vitro, are antibody-dependent cellular cytotoxicity (ADCC) (Steplewski et al, 1988; Herlyn and Koprowski, 1982) and complement-mediated cytotoxicity (Orlandi et al, 1992; Velders et al, 1994). The presence in tumours, surgically removed from MAb-treated patients, of infiltrating natural killer cells and of macrophages (Adams et al, 1984; Shetye et al, 1988) as well as of complement deposits (Adams et al, 1984), suggests that both ADCC and complement-mediated cytotoxicity may play a role in tumour cell destruction in vivo. However, as most tumour cells express increased amounts of complement-inhibiting regulators which protect the cells against lysis by autologous complement (Kumar et al, 1993; Gorter et al, 1996), the main anti-tumour mechanism of therapeutic antibodies in vivo is considered to be ADCC. The impact of antigen density and antibody affinity on the efficacy of tumour cell elimination via ADCC is relatively poorly studied, mainly because of the lack of adequate models.

In the clinical trial mentioned above (Riethmüller et al, 1994), the epithelial cell adhesion molecule (Ep-CAM) (Litvinov et al, 1994 *a,b*; 1995), a human 40-kDa epithelial glycoprotein, and one of the most frequently targeted tumour-associated antigens, was the target antigen for the murine MAb 17-1A. This MAb has been recently approved in Germany as a therapeutic reagent for the treatment of Dukes' C colorectal cancer; this indicates that Ep-CAM as a target antigen has a future in immunotherapy. Therefore, in the present study on factors defining the efficacy of ADCC, we have chosen Ep-CAM as the target antigen. Ep-CAM was targeted by MAbs 17-1A and 323/A3, which recognize overlapping epitopes on the Ep-CAM molecule and compete for binding to EP-CAM. The 323/A3 MAb affinity for Ep-CAM was found to be 40-fold higher than the affinity of MAb 17-1A (Pak et al, 1991; Velders et al, 1994; 1996). To obtain model cell lines, in which the surface expression of Ep-CAM could be varied, the Ep-CAM cDNA was introduced into Ep-CAM-negative murine and human cell lines under the transcriptional control of an inducible promoter.

*Received 7 October 1997*
*Revised 18 December 1997*
*Accepted 23 December 1997*

*Correspondence to*: SV Litvinov

**Present address:* Cardinal Bernardin Cancer Center, Loyola University Chicago, USA

The results of this study demonstrate that the efficacy and kinetics of ADCC are determined by both antibody affinity and antigen density. In addition, ADCC was shown to be dependent on LFA-1-mediated interactions, however the high-affinity MAb 323/A3 was to a lesser extent dependent on these interactions than the low-affinity MAb 17-1A.

### MATERIALS AND METHODS

### Antibodies

For all in vitro experiments with human effector cells the chimeric human/mouse versions of the antibodies 17-1A and 323/A3 were used (Sun et al, 1987; Velders et al, 1994). The chimeric antibodies contain the same human IgG1 and kappa-constant domains as both MAbs were chimerized at Centocor using identical methods and vectors. All antibodies were initially purified over protein A–Sepharose (Pharmacia, Woerden, The Netherlands), and were further purified by sequential ion-exchange chromatography on Mono S and Mono Q (Pharmacia) columns, followed by diafiltration into phosphate-buffered saline (PBS). All antibody preparations were tested using LAL assays and shown to be endotoxin negative. The anti-human CD18, clone 7E4 (Immunotech, Marseilles, France), was used in ADCC inhibition assays. To quantify the MAb binding to Ep-CAM using flow cytometry, the Fab fragments of the chimeric 323/A3 and 17-1A MAbs, directly labelled with FLUOS (Boehringer Mannheim, Mannheim, Germany), were used.

### Cell lines

The SV40 immortalized human breast epithelial cell line HBL 100, clone HCA (kindly provided by Dr J Hilkens, NKI, Amsterdam, The Netherlands), the mouse mammary carcinoma cell line L153S (Litvinov et al, 1994a) and the transfectants HCA-M and C1.5 and C1.13 were all cultured in Dulbecco's modified Eagle medium (DMEM) supplemented with 10% heat-inactivated fetal calf serum (Gibco-BRL, Paisley, UK). 50 μg ml$^{-1}$ streptomycin and 50 μg ml$^{-1}$ penicillin at 37°C and 5% carbon dioxide.

### Generation of Ep-CAM transfected cells

For the generation of Ep-CAM-expressing murine L153 cells the *SmaI*/*BglII* fragment of the GA733-2 Ep-CAM cDNA (Szala et al, 1990) was subcloned into the pJ5Ω eukaryotic expression vector (Morgenstern and Land, 1990) under the transcriptional control of the dexamethasone inducible promotor of the MMTV LTR. Two clones, C1.5 and C1.13, were used for this study. For the generation of Ep-CAM-expressing human HCA cells, the Ep-CAM cDNA was cloned into the pMEP4 expression vector (Invitrogen, Leek, The Netherlands), under the control of the inducible metallothionein promoter; the Ep-CAM-transfected HCA cell line is referred to as HCA-M. Transfection of both human and murine cells was performed using DOTAP (Boehringer Mannheim) as previously described (Litvinov et al, 1994*a*). After selection with the appropriate antibiotic, Ep-CAM expression of selected clones was identified using FACS analysis with MAb 323/A3. Induction of Ep-CAM expression in HCA-M or C1.5 and C1.13 cells was obtained by 16-h cultivation of the cells in the presence of $10^{-6}$ M dexamethasone or 1–100 μM zinc sulphate respectively.

**Figure 1** Expression of Ep-CAM in clones C1.13 and C1.5 obtained by transfection of L153 murine cells (**A**) and in transfected HCA, human mammary cells (**B**). Murine cells were transfected using a vector with the MMTV promotor. Therefore, expression of Ep-CAM could be induced by adding dexamethasone (**D**) up to $10^{-6}$ M. The HCA cells contain an episomal vector with Ep-CAM cDNA under the control of the metallothionein promoter, which can be induced by adding $Zn^{2+}$ ions at various concentrations. Data presented are mean fluorescence numbers obtained with Ep-CAM detection using directly FITC-labelled Fab fragments of MAbs c17-1A (□) or 323A3 (■). The Fab fragments were added in an excess, and their binding reflects the best estimate of Ep-CAM level at the cell surface

### Antibody-binding assay

To detect the binding of the chimeric MAbs 17-1A and 323/A3 to Ep-CAM present at the surface of the tumour cells, $1 \times 10^6$ cells were incubated in 200 μl of PBS with directly fluorescein isothiocyanate (FITC)-labelled chimeric 17-1A, 323/A3 or SF25 MAbs at 100 μg ml$^{-1}$ for 1 h at 4°C. The chimeric MAb SF25 IgG1 (Takahashi et al, 1995) was used as a non-binding negative control in these assays. After washing three times with PBS at 4°C, the cells were resuspended in 300 μl of cold PBS containing 1 μg ml$^{-1}$ propidium iodide to discriminate between viable and dead cells in flow cytometry. Immediately after staining 10 000 viable cells from each sample were examined in flow cytometry for the amount of MAb bound. The results are presented as the mean fluorescence after subtraction of the background staining with chimeric MAb SF25.

### Antibody-dependent cellular cytotoxicity

One large batch of human peripheral blood lymphocytes (PBLs) from a single healthy donor was isolated by Ficoll–Isopaque centrifugation and cryopreserved until used as effector cells. The day before the ADCC assay, a vial of human PBLs was thawed and the PBLs were activated by overnight culture in the presence of 150 IU ml$^{-1}$ IL-2 (Eurocetus, Amsterdam, The Netherlands).

© Cancer Research Campaign 1998

All ADCC experiments described in this study were performed with PBLs from the same batch. Tumour cells ($1 \times 10^6$) were detached and washed twice before labelling for 90 min with 100 μCi $^{51}Cr$ in PBS (Amersham, Buckinghamshire, UK) at 37°C and 5% carbon dioxide. Cells were washed twice with 2 ml of medium and resuspended at a concentration of $1 \times 10^4$ cells $ml^{-1}$. A 50-ml aliquot of medium containing MAb was added to 100 ml of tumour cell suspension (1000 cells per well) in 96-well round-bottom microtitre plates (Greiner, Langenthal, Switzerland), and subsequently the effector cells were added in 100 μl at various effector–target cell (E/T) ratios. After 4 h incubation at 37°C and 5% carbon dioxide, 100 μl of supernatant was removed and counted for the presence of $^{51}Cr$ (ER, experimental release) in an LKB gamma-counter. The maximum release (MR) was obtained by adding 100 μl of 1% Triton X-100 to 100 μl of labelled cells plus 50 μl of medium. Background release (BR) was measured by incubating 100 μl of labelled cells with 150 μl of medium. Specific release (SR) was calculated as follows: SR = (ER – BR / MR – BR) × 100%. All assays were performed in triplicate. In experiments on the role of the accessory molecules, the effector cells were preincubated before the experiment with the MAb against CD18 (10 μg of MAb per $10^7$ PBLs in 1 ml of DMEM for 30 min at 20°C). After the incubation, the volume of the medium was adjusted to 20 ml, and the effector suspension was added to the target cells as described above. The final concentration of anti-CD18 MAb during the cytotoxicity assay was 0.2 μg $ml^{-1}$.

### RESULTS

### Inducible expression of Ep-CAM in transfected cells

Two cell clones, C1.5 and C1.13, obtained by transfection of L153S murine carcinoma cells with Ep-CAM cDNA under the control of the inducible MMTV promotor were tested for Ep-CAM expression. The expression of Ep-CAM at the surface of cells, without and 24 h after induction with $10^{-6}$ M dexamethasone, was determined by FACScan analysis (Figure 1A). Both the basic and the induced levels of Ep-CAM expression differed significantly between clones C1.5 and C1.13. Comparison of the binding of chimeric 17-1A and 323/A3 MAbs to the Ep-CAM transfected murine cells, as was determined by flow cytometry using FITC-labelled Fab fragments, showed that at equal concentrations (the data is shown for 10 μg $ml^{-1}$), MAb 323/A3 bound approximately tenfold better than MAb 17-1A to the same target cells.

Ep-CAM expression could also be up-regulated in HCA-M cells, derived from the human HCA cell line by transfection of Ep-CAM cDNA under the control of the inducible metallothionein promoter. As analysed using flow cytometry, treatment of HCA-M cells with 100 μM zinc sulphate elevated the Ep-CAM expression approximately sixfold over uninduced Ep-CAM expression levels. Induction of Ep-CAM expression did not lead to a linearly increased binding of c17-1A and c323/A3 MAbs (Figure 1B). Nevertheless, at the same antibody concentration, MAb 323/A3 bound approximately tenfold better to the HCA-M cells than MAb 17-1A.

### ADCC is determined by antigen expression levels and antibody affinity

To examine the impact of antigen expression levels on the efficacy of ADCC, the uninduced and dexamethasone-induced murine

**Figure 2** Titration for antibody binding (solid symbols) and ADCC (open symbols) of chimeric MAbs 17-1A (**A**) and 323/A3 (**B**) on dexamethasone induced (△, ▲) and uninduced (□, ■) murine C1.5 cells. MAb binding as determined by FACScan analysis is presented as mean fluorescence on the right *y*-axis. ADCC assays were carried out at an effector–target ratio of 50:1 in parallel with the MAb-binding assay. This figure represents three independently performed MAb titration experiments

C1.5 and C1.13 Ep-CAM-transfected cell lines, respectively, were used in parallel for FACscan analysis and ADCC assays (Figure 2) with activated human PBLs and chimeric c323/A3 or c17-1A MAbs. The treatment of target cells with dexamethasone did not increase the susceptibility of cells to ADCC, as was tested using a control L153S transfectant containing Ep-CAM cDNA in the pJ3Ω expression vector under the control of the constitutive SV40 promotor (data not shown). No lysis was observed for C1.5 and C1.13 cells (data not shown) by activated human PBLs in the absence of ADCC-mediating antibodies.

In the presence of antibody, the cells with induced Ep-CAM expression were killed better in ADCC with MAbs c17-1A (Figure 2A) and c323/A3 (Figure 2B) than their uninduced counterparts. Moreover, C1.5 cells, which express higher Ep-CAM levels than C1.13 cells both before and after induction of Ep-CAM (Figure 1A), were killed more efficiently with c323/A3 and c17-1A than C1.13 cells (data not shown). With c17-1A, despite demonstrable binding of the antibody to the induced murine cells at 10 μg $ml^{-1}$,

 © Cancer Research Campaign 1998

**Figure 3** Representative graph of four kinetic ADCC experiments mediated with either 10 μg ml$^{-1}$ chimeric MAb 17-1A (**A**) or 323/A3 (**B**) in the presence of human PBL at an effector–target ratio 50:1 on human HCA cells (+) and derived Ep-CAM transfectants HCA-M cells, either uninduced HCA-M 0 (●) or induced with 10 (▲) or 100 (■) μM zinc sulphate respectively. The percentage-specific $^{51}$Cr release from the cells was determined after 2, 3 and 5 h of incubation of human target and effector cells at 37°C and 5% carbon dioxide. Results of parallel experiments on binding of chimeric 17-1A and 323/A3 MAbs to these cells are presented in Figure 1

ADCC was only obtained at MAb concentrations above 10 μg ml$^{-1}$ (Figure 2A). The binding of MAb c17-1A to dexamethasone-induced C1.5 cells was similar to the binding of MAb c323/A3 to uninduced C1.5 cells (Figure 2B), resulting in similar average numbers of MAb Fc-tails from both MAbs available for Fc-receptor binding. Nevertheless, ADCC in these two instances when mediated by MAb c323/A3 was higher than lysis mediated by MAb c17-1A. These experiments, with the data presented in Figure 2A and B, show that cell lysis by ADCC is dependent on the total amount of antibody bound to the cells but also on antibody affinity.

ADCC with murine targets was performed using human effector cells. In a combination of effector and target, one may expect a decrease, if not a complete exclusion (Mentzer et al, 1986; Johnston et al, 1990), of the adhesive interactions between the effector and target cell. The impact of MAb affinity and antigen expression levels on ADCC, in the presence of accessory adhesion molecules on the target cells and the matching counterparts on the effector cells, was studied using human PBLs against transfected human HCA-M target cells with inducible Ep-CAM expression. ADCC assays (Figure 3) and FACscan analysis for Ep-CAM expression levels and MAb binding (Figure 1B) were carried out in parallel. Again, in all experiments, MAb c323/A3 consistently mediated higher lysis than MAb 17-1A (compare the lysis levels at the 4-h time point in Figure 3).

### ADCC kinetics and MAb affinity

The kinetics of the ADCC reaction was investigated using human PBLs and human HCA-M target cells with different Ep-CAM expression levels. Specific lysis (Figure 3) with MAb c17-1A was clearly related to the amount of MAb bound to the target cells. For MAb c323/A3 this was less pronounced; the elevation of antigen expression above the baseline expression seen in the HCA-M cells without induction, led to only minor increases in ADCC (Figure 3). With mAb c17-1A on induced HCA-M cells, ADCC almost reached a lysis plateau level after 1 h of incubation. Although lysis of HCA-M cells after 1 h of incubation with MAb c323/A3 was higher than with mAb c17-1A, no lysis plateau values were evident even after 5 h of incubation. The data from these experiments showed that for human target cells the kinetics of ADCC were determined by the level of antigen expression and strongly influenced by MAb affinity.

### Influence of the adhesive effector–target interactions on ADCC in relation to MAb affinity

Using human effectors and HCA transfectants with Ep-CAM induced at different levels, we investigated to what extent the additional contact adhesions between effector and target cell affect

**Figure 4** Effect of anti-CD 18 MAb on ADCC. Targets: HCA-M cells with Ep-CAM induced by 10 μM (**A, B**) or by 100 μM zinc sulphate (**C**). Antibodies used: 1 μg of chimeric 17-1A or 323/A3 (**A**) or chimeric 17-1A in designated concentrations (**B, C**). ■, lysis in the absence of anti-CD18; □, ADCC in the presence of 0.2 μg ml$^{-1}$ of anti-CD18 MAb. Note that the negative effect of anti-CD18 MAb on ADCC (□), especially when mediated by 17-1A, can be compensated by an increase in either the concentration of MAb or the antigen density on the target cells

© Cancer Research Campaign 1998

ADCC. As was tested in flow cytometry, neither ICAM-1 nor LFA-3 expression on HCA and HCA-M cells was substantially affected by the $Zn^{2+}$ cations in medium or by Ep-CAM expression (not shown). As shown in Figure 4A, at a given concentration of the MAb, ADCC mediated by a high-affinity MAb c323/A3 was not sensitive to the blocking of the effector's accessory molecules with an anti-CD18 MAb. For the low-affinity MAb c17-1A, the ADCC was blocked at the given conditions; however, by either increasing the MAb concentration 100 times (Figures 4B and 4C) or by inducing the antigen expression on the target cells (Figure 4C) MAb 17-1A mediated lysis of the targets even in the presence of the anti-CD18 MAb. For the high-affinity MAb c323/A3 the observations were similar, but at more than 100 times lower concentrations of the MAb (data not shown). These results suggest that, except for MAb affinity and antigen density, the efficacy of ADCC also depends on the presence of accessory molecules. However, the absence of additional effector–target adhesive interactions may be compensated by increasing the number or strength of the MAb-mediated interactions between effector and target cells.

### DISCUSSION

To be effective, immunotherapy of tumours requires complete elimination of tumour cells. At present, it is not known to what extent heterogeneous antigen expression by individual tumour cells represents a problem in therapy with MAbs, but it is quite likely that tumour cells with low levels of target antigen, similarly to the antigen-negative cells, may escape MAb-mediated immunotherapy (Fleuren et al, 1995). As ADCC is considered to be the major mechanism of MAb action in therapy (Adams et al, 1984), we performed this study on the importance of antigen density and MAb affinity for ADCC efficacy. In contrast to other studies in this field (Capone et al, 1984; Hagan et al, 1986; Fogler et al, 1988), our approach is the first one that allows the study of the effect of antigen density on ADCC on established tumour cell clones with inducible antigen expression.

The results showed that the antibody affinity was of major importance for effective tumour cell binding and lysis through ADCC as the high-affinity MAb 323/A3, at equal MAb concentrations, bound better to tumour cells with the same antigen expression than the low-affinity MAb 17-1A. Even at comparable levels of binding to the targets the 323/A3 MAb was more efficient than 17-1A. In addition, it was found that the efficacy of ADCC with both MAbs 17-1A and 323/A3 was dependent on Ep-CAM expression levels both in murine and in human model cell lines. Cells with relatively higher Ep-CAM levels were lysed more efficiently than cells with lower Ep-CAM expression levels.

As a consequence, MAb 323/A3 consistently mediated higher ADCC than MAb 17-1A against the same tumour cells. This is in agreement with our previous results on Ep-CAM expressing LS 180 human colorectal carcinoma cells (Velders et al, 1995). Moreover, not only in vitro, but most importantly also in vivo, MAb 323/A3 mediated killing of tumour cells that were not killed by MAb 17-1A (Velders et al, 1996). In agreement with Herlyn et al. (1985), our results, as presented in Figure 2, show that ADCC requires a minimal threshold number of antibodies bound to the target cells and that this threshold is dependent on antibody affinity, i.e. higher for a low-affinity MAb such as 17-1A than for a high-affinity MAb 323/A3. Against murine tumour cells with low Ep-CAM expression levels (see Figure 2) chimeric MAb 17-1A was unable to elicit a cytotoxic response at MAb concentrations below 50 $\mu g\ ml^{-1}$, whereas MAb 323/A3 at 1 $\mu g\ ml^{-1}$ effectively killed these low Ep-CAM-expressing cells. Apparently not only the number of engaged $Fc R\gamma$ receptors on the effector cell is important, but also the strength of the interconnections to the target antigen affects the activation signal to the effectors (Velders et al, 1996)

The requirement of accessory adhesions between effector and target for ADCC is not very clear, however the scope of the publications on the subject suggests their importance (Liesveld et al, 1991; Webb et al, 1991; Edwards et al, 1992; Kushner and Cheung, 1992). Our results suggest that the importance of additional adhesive interactions between effector and target cells in ADCC is influenced by the overall number and strength of antibody-mediated interconnections between the two cells. As both antibodies used, 17-1A and 323/A3, have identical Fc fragments [owing to their chimerization using the same genes for the constant Ig chains (Sun et al, 1987; Velders et al, 1994)], it is the affinity of the antibody to the target antigen or the three-dimensional orientation of Fc tails that is of importance. From the results presented in Figure 4 we conclude that high MAb affinity, or a large number of antibody–antigen interconnections between effector and target cells, may well compensate the reducing effect on ADCC of the suppressed accessory interactions.

Knowledge of the kinetics of the ADCC reaction with different MAbs could also help when choosing MAbs for immunotherapy. We provided evidence that, against the same HCA-M cells, the high-affinity MAb 323/A3 not only mediated higher lysis, but also continued to mediate cell lysis for an extended period, whereas an extended incubation with MAb 17-1A had no additional effect. The observed lack of plateau values in ADCC in a 5 h assay against human target cells with MAb 323/A3, and the overall higher lysis levels obtained, indicates that MAb 323/A3 can mediate the killing of Ep-CAM-expressing tumour cells that cannot be killed by MAb 17-1A, which is in agreement with our previous in vivo results (Velders et al, 1996). It seems plausible that only the cells with the highest levels of Ep-CAM expression were killed by the 17-1A MAb, and that cells with lower levels of expression were not lysed. However, such cells could be lysed by the 323/A3 MAb. Based on our previous in vivo results and the data presented here, we conclude that the high-affinity 323/A3 MAb can eliminate substantially more cells from a heterogeneous tumour cell population than the low-affinity MAb 17-1A. It's also possible that low Ep-CAM-expressing tumour cells in patients may be more effectively eradicated using the high-affinity MAb 323/A3 instead of MAb 17-1A.

### ABBREVIATIONS

ADCC, antibody dependent cellular cytotoxicity; Ep-CAM, epithelial cell adhesion molecule; i.p., intraperitoneal; s.c., subcutaneous; LTR, long terminal repeat; MAb, monoclonal antibody; MMTV, murine mammary tumour virus; PBLS, peripheral blood lymphocytes.

### ACKNOWLEDGEMENTS

The authors would like to thank Dr Bolhuis for critically reading the manuscript. This research was performed with financial support of the Technology Foundation STW (grant LB100.2398) and the Dutch Cancer Society (grant RUL 94-762).

© Cancer Research Campaign 1998

### REFERENCES

Adams DO, Hall T, Steplewski Z and Koprowski H (1984) Tumors undergoing rejection induced by monoclonal antibodies of the IgG2a isotype contain increased numbers of macrophages activated for a distinctive form of antibody-dependent cytolysis. *Proc Natl Acad Sci USA* **81**: 3506–3510

Capone PM, Papsidero LD and Chu TM (1984) Relationship between antigen density and immunotherapeutic response elicited by monoclonal antibodies against solid tumors. *J Natl Cancer Inst* **72**: 673–677

Edwards BS, Nolla HA and Hoffman RR (1992) Resolution of adhesion- and activation-associated components of monoclonal antibody-dependent human NK cell-mediated cytotoxicity. *Cell Immunol* **144**: 55–68

Fleuren GJ, Gorter A, Kuppen PJK, Litvinov SV and Warnaar SO (1995) Tumor heterogeneity and immunotherapy of cancer. *Immunol Rev* **145**: 91–122

Fogler WE, Klinger MR, Abraham KG, Gottlinger HG, Riethmüller G and Daddona PE (1988) Enhanced cytotoxicity against colon carcinoma by combinations of noncompeting monoclonal antibodies to the 17-1A antigen. *Cancer Res* **48**: 6303–6308

Gorter A, Block VT, Haasnoot WHB, Ensink NG, Daha MR and Fleuren GJ (1996) Expression of CD46, CD55, and CD59 on renal tumor cell lines and their role in preventing complement-mediated tumor cell lysis. *Lab Invest* **74**: 1039–1049

Hagan PL, Halpern SE, Dillman RO, Shawler DL, Johnson DE, Chen A, Krishnan L, Frincke J, Bartholomew RM and David GS (1986) Tumor size: effect on monoclonal antibody uptake in tumor models. *J Nucl Med* **27**: 422–427

Herlyn D and Koprowski H (1982) IgG2a monoclonal antibodies inhibit human tumor growth through interaction with effector cells. *Proc Natl Acad Sci USA* **79**: 4761–4765

Herlyn D, Powe J, Ross AH, Herlyn M and Koprowski H (1985) Inhibition of human tumor growth by IgG2a monoclonal antibodies correlates with antibody density on tumor cells. *J Immunol* 134: 1300–1304

Johnston SC, Dustin ML, Hibbs ML and Springer TA (1990) On the species specificity of the interaction of LFA-1 with intercellular adhesion molecules. *J Immunol* **145**: 1181–1187

Kumar S, Vinci JM, Pytel BA and Baglioni C (1993) Expression of messenger RNAs for complement inhibitors in human tissues and tumors. *Cancer Res* **53**: 348-353

Kushner BH and Cheung NK (1992) Absolute requirement of CD11/CD18 adhesion molecules, FcRII and the phosphatidylinositol-linked FcRIII for monoclonal antibody-mediated neutrophil antihuman tumor cytotoxicity. *Blood* **79**: 1484–1490

Liesveld JL, Frediani KE, Winslow JM, Duerst RE and Abboud CN (1991) Cytokine effects and role of adhesive proteins and Fc receptors in human macrophage-mediated antibody dependent cellular cytotoxicity. *J Cell Biochem* **45**: 381–390

Litvinov SV (1995) Ep-CAM: a homophilic cell–cell adhesion molecule with EGF-like domains. *Trends Glycosci Glycotechn* **7**: 261–271

Litvinov SV, Velders MP, Bakker HAM, Fleuren GJ and Warnaar SO (1994*a*) Ep-CAM: a human epithelial antigen is a homophilic cell–cell adhesion molecule. *J Cell Biol* **125**: 437–446

Litvinov SV, Bakker HA, Gourevitch MM, Velders MP and Warnaar SO (1994*b*) Evidence for a role of the epithelial glycoprotein 40 (Ep-CAM) in epithelial cell–cell adhesion. *Cell Adhes Commun* **2**: 417–428

Mentzer SJ, Barbosa JA, Strominger JL, Biro PA and Burakoff SJ (1986) Species-restricted recognition of transfected HLA-A2 and HLA-B7 by human CTL clones. *J Immunol* **137**: 408–412

Morgenstern JP and Land H (1990) A series of mammalian expression vectors and characterisation of their expression of a reporter gene in stably and transiently transfected cells. *Nucleic Acids Res* **18**: 1068

Orlandi R, Figini M, Tomassetti A, Canevari S and Colnaghi MI (1992) Characterization of a mouse–human chimeric antibody to a cancer-associated antigen. *Int J Cancer* **52**: 588–593

Pak KY, Nedelman MA, Fogler WE, Tam SH, Wilson E, Van Haarlem LJ, Colognola R, Warnaar SO and Daddona PE (1991) Evaluation of the 323/A3 monoclonal antibody and the use of technetium-99m-labeled 323/A3 Fab′ for the detection of panadenocarcinoma. *Int J Rad Appl Instrum* **18**: 483–497

Riethmüller G, Schneider-Gadicke E, Schlimok G, Schmiegel W, Raab R, Hoffken K, Gruber R, Pichlmaier H, Hirche H and Pichlmayr R (1994) Randomised trial of monoclonal antibody for adjuvant therapy of resected Dukes' C colorectal carcinoma. *Lancet* **343**: 1177–1183

Shetye J, Frodin JE, Christensson B, Grant C, Jacobsson B, Sundelius S, Sylven M and Mellstedt H (1988) Immunohistochemical monitoring of metastatic colorectal carcinoma in patients treated with monoclonal antibodies (MAb 17-1A). *Cancer Immunol Immunother* **27**: 154–162

Steplewski Z, Sun LK, Shearman CW, Ghrayeb J, Daddona P and Koprowski H (1988) Biological activity of human-mouse IgG1, IgG2, IgG3, and IgG4 chimeric monoclonal antibodies with antitumor specificity. *Proc Natl Acad Sci USA* **85**: 4852–4856

Sun LK, Curtis P, Rakowicz Szulczynska E, Ghrayeb J, Chang N, Morrison SL and Koprowski H (1987) Chimeric antibody with human constant regions and mouse variable regions directed against carcinoma-associated antigen 17-1A. *Proc Natl Acad Sci USA* **84**: 214–218

Szala S, Froehlich M, Scollon M, Kasai Y, Steplewski Z, Koprowski H and Linnenbach AJ (1990) Molecular cloning of cDNA for the carcinoma-associated antigen GA733-2. *Proc Natl Acad Sci USA* **87**: 3542–3546

Takahashi H, Nakada T, Nakaki M and Wands JR (1995) Inhibition of hepatic metastases of human colon cancer in nude mice by a chimeric SF-25 monoclonal antibody. *Gastroenterology* **108**: 172–182

Velders MP, Litvinov SV, Warnaar SO, Gorter A, Fleuren GJ, Zurawski VR Jr and Coney LR (1994) New chimeric anti-pancarcinoma monoclonal antibody with superior cytotoxicity-mediating potency. *Cancer Res* **54**: 1753–1759

Velders MP, van Rhijn CM, Briaire IH, Fleuren GJ, Warnaar SO and Litvinov SV (1995) Immunotherapy with low and high affinity monoclonal antibodies 17-1A and 323/A3 in a nude mouse xenograft carcinoma model. *Cancer Res* **55**: 4398–4403

Velders MP, van Rhijn CM, Cornelissen IMHA, van Muijen GNP, Briaire IH, Fleuren GJ, Warnaar SO and Litvinov SV (1996) The role of antibody affinity in tumor immunotherapy evaluated in in vivo models for minimal residual disease. *J Immunother* **19**: 245–256

Webb DSA, Mostowski HS and Gerrard TL (1991) Cytokine-induced enhancement of ICAM-1expression results in increased vulnerability of tumor cells to monocyte-mediated lysis. *J Immunol* **146**: 3682–3686

© Cancer Research Campaign 1998